# Intracranial extension of adenoid cystic carcinoma: potential involvement of EphA2 expression and epithelial-mesenchymal transition in tumor metastasis: a case report

**DOI:** 10.1186/1756-0500-7-131

**Published:** 2014-03-07

**Authors:** Junya Fukai, Koji Fujita, Toshikazu Yamoto, Takahiro Sasaki, Yuji Uematsu, Naoyuki Nakao

**Affiliations:** 1 Department of Neurological Surgery, Wakayama Medical University School of Medicine, Kimiidera 811-1, Wakayama 641-0012, Japan; 2 School of Health and Nursing Science, Wakayama Medical University, Mikazura 588, Wakayama 641-0011, Japan

**Keywords:** Adenoid cystic carcinoma, Migration, Invasion, Metastasis, EphA2, Epithelial-mesenchymal transition

## Abstract

**Background:**

Adenoid cystic carcinoma is a malignant epithelial tumor derived from salivary glands and tends to invade the surrounding structures including nervous system. We present a case of adenoid cystic carcinoma with intracranial extension and propose a novel molecular mechanism of adenoid cystic carcinoma metastasis.

**Case presentation:**

A 29-year-old Japanese male presented with left trigeminal nerve disturbance. Neuroimaging revealed a tumor located at the right middle cranial and infratemporal fossa. The tumor was removed via a subtemporal extradural and infratemporal fossa approach and histologically diagnosed as adenoid cystic carcinoma. Radiological and operative findings confirmed a perineural spread of the tumor along the mandibular nerve. Immunohistochemical analyses of molecular consequences in this case were performed for better understanding of the biological processes associated with adenoid cystic carcinoma metastasis. First, the neoplastic cells were not immunoreactive for E-cadherin, an epithelial marker, but for vimentin, a mesenchymal marker, suggesting changes in cell phenotype from epithelial to mesenchymal states. Correspondingly, immunoreactivity of transcriptional factors, such as Slug, Twist, matrix metalloproteinase-2 and -9, which are involved in epithelial–mesenchymal transition, were observed. Second, elevated expression of EphA2 receptor, not ephrin-A1, was notable in the neoplastic cells, suggesting morphological changes reminiscent of epithelial–mesenchymal transition and ligand-independent promotion of tumor cell migration and invasion.

**Conclusions:**

We report a case of adenoid cystic carcinoma with perineural spread and provide the first published evidence that EphA2 expression without ephrin-A1 and epithelial–mesenchymal transition might play important roles in adenoid cystic carcinoma progression.

## Background

Adenoid cystic carcinoma (ACC), a malignant epithelial neoplasm, is a rare tumor entity and comprises about 1% of all malignant tumor of the oral and maxillofacial region [1]. It is a slowly growing but highly invasive cancer with high recurrence rate. Intracranial ACC even is more rare and has been reported as 4 – 22% of ACC [2]. It could be primary or secondary which could occur either by direct invasion, hematogenous spread, or perineural infiltration [1]. The most common route is via contiguous perineural invasion [2]. Many authors describe worse prognosis for ACC of the minor salivary glands, due to early local infiltration and invasion of surrounding tissue and bone [1]. However, molecular pathology of the high invasive and metastatic potential of ACC is not well understood. Better understanding of the biological processes associated with ACC invasion and metastasis could provide a new approach to improve prognosis of the patients with ACC.

The epithelial–mesenchymal transition (EMT) is the conversion of cells with an epithelial phenotype into cells with a highly motile fibroblastoid or mesenchymal phenotype [3]. Diverse signaling pathways can regulate an EMT program [4]. The process of EMT is characterized by loss of intercellular adhesion and polarity, cytoskeletal reorganization that enhances cell motility, and degradation of the basement membrane. EMT is a critical mechanism in embryonic development, chronic inflammation and fibrosis, and is considered to play a key role in tumor progression and metastasis [4]. Therefore, it is speculated that EMT could be involved in ACC invasion and metastasis.

The Eph receptors represent the largest family of receptor protein tyrosine kinases and interact with their ligands, ephrins [5]. Eph-ephrin signaling is fundamentally involved in developmental processes or in the remodeling of blood vessels and mainly affects the cell shape and motility by regulating cytoskeletal organization and cell adhesion, and also influences cell proliferation and cell-fate determination [6]. So far, the genes for Eph receptors and ephrins have been recognized to be differentially expressed in various human tumors [5]. Previous bodies of evidence have implicated Eph involvement in the altered tumor behavior such as increased invasiveness or increased metastatic potential, and consequently with poor patient outcome [5]. Therefore, it is supposed that Eph signaling could play some roles in tumor progression of ACC as one of their possible consequences.

In this article, we report a case of ACC arising in the infratemporal fossa and extending into the middle cranial base through the foramen ovale along the mandibular nerve. With this case, molecular pathology of ACC invasion and metastasis is also discussed, focusing on EMT program and EphA2 signaling as a possible mechanism.

## Materials and methods

### Clinical sample

The protocol is in compliance with the Helsinki Declaration and was ethically approved by the institutional review board at Wakayama Medical University (Permit number: 61). Fresh tumor tissue and written informed consent were obtained from the patient

### Antibodies

Antibodies were purchased as follows: rabbit monoclonal to E-cadherin (3195, Cell Signalling, Boston, MA, USA; dilution, 1:400) and Slug (9585, Cell Signalling; dilution, 1:100), mouse monoclonal to Vimentin (m0725, DakoCytomation, Kyoto, Japan: dilution, 1:2500), Twist (ab50887, Abcam, Cambridge, UK; dilution, 1:250) and matrix metalloproteinase-2 (MMP-2) (MS804, ThermoFisher Scientific, Cheshire, UK; dilution, 1:100) and rabbit polyclonal to EphA2 (sc-924, Santa Cruz Biotechnology, CA USA; dilution, 1:50), ephrinA1 (sc-20719, Santa Cruz Biotechnology; dilution, 1:50), MMP-9 (RB9234, ThermoFisher Scientific; dilution, 1:500).

### Immunohistochemistry

Immunohistochemistry was carried out using the VECTASTAIN® ABC kit (Vector Laboratories, Burlingame, CA, USA). Briefly, paraffin sections were deparaffinized in xylene and rehydrated through a graded series of ethanol dilutions. Endogenous peroxidase activity was blocked by incubation in 0.3% hydrogen peroxidase for 30 minutes. After pretreatments performed according to manufacturer’s instructions, sections were incubated at room temperature for 1 h with primary antibody, and visualized with diaminobenzidine. Diaminobenzidine was used as the final chromogen. Immunostained cells were examined under a microscope (Keyence Biozero, Osaka, Japan).

### Reverse transcription polymerase chain reaction (RT-PCR)

After washing twice with ice-cold PBS, 1 mL of IsoGene (Nippongene, Toyama, Japan) reagent was added to an excised tissue. Sample was stored at -80°C. Total ribonucleic acid (RNA) was isolated following the IsoGene protocol as described by the manufacturer. Commercially available total RNA of human normal salivary gland (BioChain Institute, Inc., SF, USA) was used as a control. *EphA2* messenger RNA (mRNA) expression was analyzed by RT-PCR with TaKaRa RNA PCR Kit (AMV) Ver.3.0 (TakaraBio, Shiga, Japan) also as instructed. RT-PCR for *glyceraldehyde-3-phosphate dehydrogenase* (*GAPDH*) served as an internal control. Primer sequences for human *EphA2* (369 bp) and *GAPDH* (408 bp) were 5'- GCA GGA GAG TTT GGG GAG GTG T-3’ & 5'- GGT TGC TGT TGA CGA GGA TGT T-3’ and 5'-CCC ATC ACC ATC TTC CAG GAG-3’ & 5'-AGG GAT GAT GTT CTG GAG AGC C- 3', respectively.

## Case presentation

**Figure 1:**
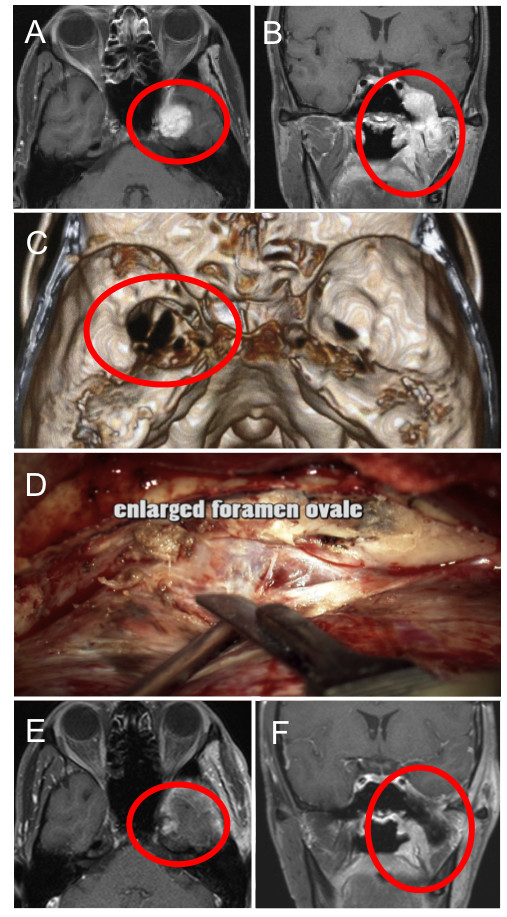
**Pre-, Intra-, and Post-operative findings of the present case. A** and **B**, Gadolinium-enhanced T1-weighted magnetic resonance images revealing a mass in the infra-temporal and middle cranial fossa (circle). **C**, Three-dimensional bone image computed tomography scan demonstrating widening of the foramen ovale (circle). **D**, Intra-operative finding showing the tumor and enlarged foramen ovale. **E** and **F**, Postoperative gadolinium-enhanced T1-weighted magnetic resonance images manifesting removal of the tumor (circle).

**Figure 2:**
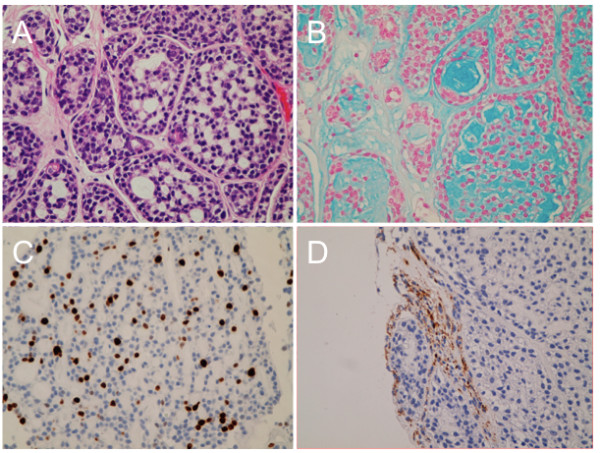
**Histopathological findings of the present case. A**, Photomicrographs of the tumor specimen showing a moderately cellular tumor composed of compact epithelial cells showing tubular pattern. Hematoxylin & Eosin. Original magnification × 200. **B**, Alcian-blue stain revealing myxoid or colloid materials in the tubules. **C**, Molecular immunology Borstel-1 (MIB-1) staining index indicating around 10%. **D**, Immunohistochemistry for neurofibrillary protein demonstrating neural bundles in the tumor.

A previously healthy 29-year-old Japanese man presented with numbness of the left face that progressed over the next month, followed by chewing difficulty. A physical examination revealed left fifth cranial nerve palsy. Computed tomography scanning of the head demonstrated a mass in the left middle cranial fossa and widening of the foramen ovale (Figure 1). Magnetic resonance imaging revealed the mass involving the left infra-temporal and middle cranial fossa (Figure 1). The mass was removed via a subtemporal epidural and infra-temporal fossa approach; after total removal of the mass in the middle fossa, the dilated foramen ovale was visible, and an infratemporal fossa mass was removed as much as possible (Figure 1). Intraoperative findings supposed the infratemporal fossa tumor extending intracranially along the mandibular nerve. Histological examination revealed a moderately cellular tumor composed of compact epithelial cells showing tubular pattern surrounded by fibrovascular septa (Figure 2). In differentiated tubules, myxoid and colloid materials were contained. Degenerative neural bundles were also seen, suggesting a perineural invasion. The tumor cells were immunoreactive for cytokeratin AE1/AE3. Molecular immunology Borstel-1 (MIB-1) staining index in the solid form were around 10%, suggesting higher grade than typical ACC. This pathological analysis indicated ACC of the minor salivary glands. The patient underwent fractionated irradiation of the lesion.

### Molecular pathology

**Figure 3:**
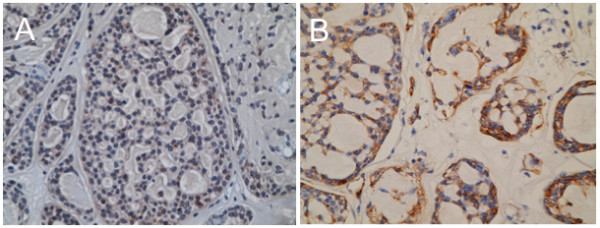
**Epithelial-mesenchymal transition in the present case.** Immunohistochemistry for E-cadherin **(A)** and vimentin **(B)**. Note that E-cadherin **(A)**, an epithelial marker, is almost negative in the tumor cell, while vimentin **(B)**, a mesenchymal marker, is positive.

**Figure 4:**
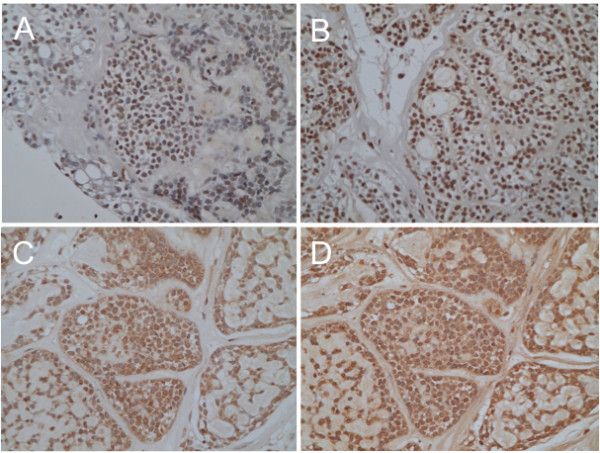
**Epithelial-mesenchymal transition in the present case.** Immunohistochemistry for transcriptional factors, Slug **(A)** and Twist **(B)**. Nuclear staining for Slug and Twist was observed in the neoplastic cells. Immunohistochemistry for mesenchymal markers, matrix metalloproteinase-2 **(C)** and -9 **(D)**. Both matrix metalloproteinase-2 **(C)** and -9 **(D)** are expressed in the tumor cells.

**Figure 5:**
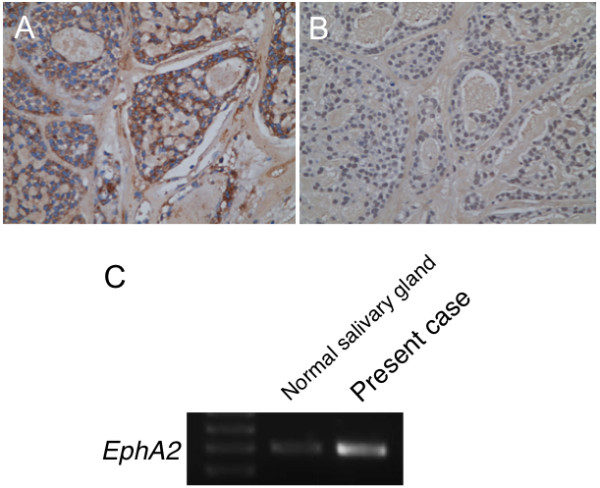
**EphA2 and ephrinA1 expression in the present case.** Immunohistochemistry for EphA2 **(A)** and ephrinA1 **(B)**. Note that EphA2 **(A)** staining is positive in the tumor cell, while ephrinA1 **(B)** is negative. Original magnification × 200. **(C)**, *EphA2* was identified in the frozen tissue by reverse transcription-PCR, with higher mRNA expression in the tumor than normal salivary gland.

For better understanding of the biological factors associated with ACC metastasis, we performed immunohistochemical analyses. First, we hypothesized that EMT might be involved in the metastatic process of ACC, because accumulating evidence suggest that epithelial tumor cells can switch from a sessile, epithelial phenotype to a motile, mesenchymal phenotype referred to as EMT, resulting in tumor migration and metastasis [7]. The loss of typical epithelial cell markers, such as E-cadherin, and the gain of mesenchymal markers, such as vimentin, are hallmarks of EMT [7]. Indeed, the neoplastic cells were immunoreactive for E-cadherin, but not for vimentin in the present case (Figure 3). Correspondingly, immunoreactivity of transcriptional factors, such as Slug, Twist, MMP-2 and MMP-9, which have been reported to be involved in EMT, were observed (Figure 4). These findings suggest that EMT might be induced in this case, although it remains unclear which factor could induce this event. Second, we suspected the involvement of aberrant expression of Eph receptor tyrosine kinases and their ephrin ligands in the ACC metastasis, because Eph receptors and ephrins have been shown to affect the migration and invasion of cancer cells in culture as well as tumor invasiveness and metastasis *in vivo*[5]. In the present case, higher staining intensity for EphA2 receptor, not for ephrin-A1, was notable in the neoplastic cells (Figure 5). For reference, the *EphA2* were identified in ACC frozen tissues by RT-PCR, with higher mRNA expression in ACC tissues than normal salivary glands (Figure 5). From these findings, it was revealed that elevated expression of EphA2 without ephrin-A1 might occur in this case. Taken together, our immunohistochemical analyses suggest that EphA2 signaling and EMT program might be associated with ACC metastatic process.

## Discussion

We report a case of ACC arising from the minor salivary gland and extending into the middle cranial fossa along the mandibular nerve. In this case, it is considered that ACC perineurally spread from extracranial to intracranial space, in that ACC is characterized by extensive migration and invasion along basement membrane-rich tissues, such as perineural and vascular sheaths. ACC is the well-known malignancy with a propensity for perineural invasion, whereas the molecular mechanism behind metastatic process remains unclear. Thus we performed an immunohistochemical analysis demonstrating that overexpression of EphA2 and induction of EMT occurred in the tumor cells of this case. These findings suggest that EphA2 signaling and EMT program might be potentially involved in invasive and metastatic potential of ACC.

Acquisition of an invasive phenotype of cancer cells in primary tumors is an absolute requirement for metastasis [7]. A process that causes a functional phenotypic transition of polarized epithelial state into migratory mesenchymal state, referred to as EMT, should be necessary. Our observation of decreased expression of E-cadherin, an epithelial cell marker, in the immunohistochemistry of the present case revealed a loss in epithelial characteristics of cancer cells. Transcriptional factors, Slug and Twist, expression and nuclear translocation were observed, leading to potential repression of E-cadherin and induction of EMT. Then, expression of vimentin and MMPs represents cytoskeletal reorganization that enhances cell motility and proteinase secretion for degradation of the basement membrane, respectively, frequently considered to acquire mesenchymal features. Recently, Ishii *et al.* revealed that EMT is involved in the ACC metastasis by means of a DNA microarray analysis using their ACC cell lines [8]. Taken together, it appears that the EMT is a key developmental program that is often activated during ACC invasion and metastasis.

The Eph receptors and ephrins are divided into two subclasses, A and B, based on their homologies, structures, and binding affinities [5]. Fourteen Eph receptors and eight ephrin ligands have been identified so far in mammals [5]. In the previous reports, one family member, EphA2, is strongly overexpressed in many types of tumor, such as glioblastomas, ovarian cancers and prostate adenocarcinomas, and the increased expression of EphA2 correlates with enhanced metastatic potential and poor prognosis [9]. Here, we determined high expression of EphA2 in clinical specimens of ACC in both protein and mRNA levels. Noticeably, ephrinA1 was otherwise expressed at low levels when EphA2 was overexpressed in our case, as the expression pattern of ephrinA1 in some tumors was documented different from EphA2 [9]. On the other hand, there is a recent study showing that not only EphA2 but also ephrinA1 was highly expressed in adenoid cystic carcinoma and correlated with tumor invasion [10]. In this study, the authors also described that EphA2 was present in a non-tyrosine-phosphorylated state despite of high expression of ephrin-A1. In terms of this point, Miao and his colleagues reported on the diametrically opposite roles of EphA2 in regulating cell migration and invasion; while activation of EphA2 with ephrin-A1 inhibited migration of cancer cells, EphA2 overexpression promoted migration in a ligand-independent manner [11]. On the basis of this evidence, our observation of high expression of EphA2 without ephrinA1 is a reasonable finding for explanation of high invasive and metastatic potential of ACC. Presumably EphA2 might play a role in ACC metastasis independent of the presence of ephrin-A1. Anyway it was worth to further investigate the function of EphA2 in ACC cell migration and invasion.

We provide the evidence that expression of EphA2 and induction of EMT have occurred in a case of ACC metastasis and discuss the possibility that EphA2 and EMT are involved as a mechanism of ACC metastasis. However, it remains unclear whether the EphA2 signaling could induce EMT program. A number of EMT inducers have been identified that include growth factors, cytokines and microRNAs, and diverse signaling pathways have been reported to regulate EMT [7]. In the literature, EphA2 signaling in epithelial and cancer cells can induce morphological changes reminiscent of transition between epithelial and mesenchymal states [5]. Concretely, stimulation of EphA2 forward signaling with ephrin-A1 enhances the maturation of cell–cell junctions and cell compaction, leading to an epithelial phenotype [5]. Moreover, E-cadherin can promote EphA2 expression and surface localization in epithelial and cancer cells, thereby prolonging EphA2 interaction with ephrin-A1 [5]. Therefore, it may be possible that, without ephrin-A1 stimulation, EphA2 overexpression and forward signaling down-regulate E-cadherin in a feedback loop, inducing a phenotypic change from epithelial to mesenchymal character; however, it remains to be elucidated and further investigation should be needed.

## Conclusions

Finally, the results of our study revealed that EMT might be involved in the ACC metastatic process. We also showed that the expression of EphA2, without ephrinA1, is elevated in our case of ACC with intracranial extension. Although *in vitro* and i*n vivo* experiments should be necessary to elucidate whether aberrant EphA2 signaling could induce EMT program, resulting in invasion and metastasis of ACC, targeting these processes might be an attractive but challenging approach that is likely to lead to improved clinical management of ACC patients.

## Consent

Written informed consent was obtained from the patient for publication of this case report and any accompanying images. A copy of the written consent is available for review by the Editor-in-chief of this journal.

## Abbreviations

ACC: Adenoid cystic carcinoma; EMT: Epithelial–mesenchymal transition; RT-PCR: Reverse transcription polymerase chain reaction; MMP: Matrix metalloproteinase; RNA: Ribonucleic acid; mRNA: Messenger RNA; GAPDH: Glyceraldehyde-3-phosphate dehydrogenase.

## Competing interests

The authors declare that they have no competing interests.

## Authors’ contributions

JF carried out the whole study and drafted the manuscript. KF, TY, and TS acquired the clinical data. YU performed the histopathological analysis. NN directed the study. All authors read and approved the final manuscript.
